# A comparison of three organisational levels in one health care region in Sweden implementing person-centred care: coupled, decoupled or recoupled in a complex organisation

**DOI:** 10.1186/s12913-022-07548-8

**Published:** 2022-02-14

**Authors:** Malin Tistad, Lars Wallin, Eric Carlström

**Affiliations:** 1 School of Health and Welfare, Dalarna University, SE 791 88 Falun, Sweden; 2 Department of Neurobiology, Care Sciences and Society, Karolinska Institutet, Stockholm, Sweden; 3 Centre for Person-Centred Care (GPCC), University of Gothenburg, Gothenburg, Sweden; 4 Institute of Health and Care Sciences, Sahlgrenska Academy, University of Gothenburg, Gothenburg, Sweden

**Keywords:** Implementation, Spread, Person-centred care, Health policy, Sweden

## Abstract

**Background:**

Establishing more substantial patient involvement in the health care has become fundamental to Western health care services. Person-centred care (PCC) has been developed as a way of working that involve the patients and family members. However, the implementation of PCC in clinical practice has proven to be challenging. The aim of this study was to explore the congruence of managers’ perceptions and understanding of various aspects of PCC across three organisational levels in one health care region in Sweden in terms of coupling, decoupling and recoupling.

**Methods:**

A policy on increased patient participation in health care was adopted in one health care region in Sweden. This policy was embodied in the form of PCC and a support strategy for the implementation was put in place. Participants representing three organisational levels (*senders:* politicians, *n* = 3; *messengers:* senior management, *n* = 7; and *receivers:* middle- and frontline managers, *n* = 13) were interviewed and documents collected. A deductive qualitative content analysis was performed and findings from the three organisational levels compared.

**Results:**

Descriptions of PCC at all the three organisational levels included health care provided in partnership between provider and patient. However, messengers and receivers also included aspects of how work was organised as part of the concept. Representatives at all levels expected high-quality care while reducing health care costs as an outcome, however, messengers and receivers also anticipated improvements in the work environment and reduced staff turnover. Strategies to support implementation included continuation and enhancement of existing routines that were considered person-centred and development of new ones. A need to make PCC less ‘fuzzy’ and ambiguous and instead communicate a more tangible care process was described. Representatives among messengers and receivers also suggested that no actions were needed because the practice was already considered person-centred.

**Conclusion:**

The findings indicated that congruence between organisational levels existed in some aspects, suggesting coupling between policy and practice. However, also incongruences were identified that might be due to the fuzziness of definitions and the application of PCC in practice, and the difficulty in assessing the level of patient-centredness in clinical practice.

**Supplementary Information:**

The online version contains supplementary material available at 10.1186/s12913-022-07548-8.

## Background

New health care concepts have emerged as an effect of modern technology, social structures and beliefs. The health care sector has developed from firm hierarchical structures of power and staff superiority [1] to a service that should be tailored to the needs and preferences of the patient. Healthcare organisations have discerned the need to manage diversity, involve the patient in a co-production process, customise the care and improve patient satisfaction and service quality [2, 3]. A comparative study shows that Sweden, Australia and the United Kingdom have issues coordinating health care providers [4]. And when comparing European countries, Sweden is one of the countries in which patients experience a greater lack of continuity and involvement in care [4, 5]. Such studies put pressure on stakeholders to improve health care services in accordance to socio-political equity [6].

Establishing more substantial patient involvement in the delivery of health care has become fundamental to health reforms across Western health care services [7]. Even though patient satisfaction, proximity, accessibility and response times still need to be improved, the status of the patient has changed [8]. Critiques from patient advocacy groups and civil rights movements towards health services have been raised [3] and, as an effect, patients have become more involved and make a difference in several stages of health care and care planning [9].

The willingness to listen and adapt health services according to the patient’s needs has been the driving force when creating legislation to defend the autonomy and rights of the patient. In most western countries patient participation has increasingly been recognised as a key component in legislation regulating the health care processes [10]. The World Health Organisation (WHO) and the World Alliance for Patient Safety are actively highlighting the role that patients and their families should play in the health care sector [11]. According to Swedish law, patients should have a central position in planning and coordinating their care and in accessing information such as health record data [12].

Even though staff, care teams and organisations show interest and even commitment to involving patients and family members, they may lack clarity about who to involve and the goals of involvement [13]. To provide ways of working for involving patients and family members, several concepts have been developed including person-centred care (PCC), patient-centred care, family-centred care, child-centred care, client-centred care and relationship-centred care [14–18]. Accordingly, the PCC approach has been brought forward in Swedish national policy [19] and there is a strong movement towards its adoption in the Swedish health care regions [20].

### Theoretical framework

The concepts of coupling, decoupling and recoupling in a complex organisation are examined in this study. Coupling takes place when formal policies and actual organisational practices are connected and in congruence with one another. In idealistic models of management control a top-down coupling is when policies are distributed from management and applied into actions by subordinates. In contrast, decoupling refers to gaps between policies and actual practices [21]. The gaps are situated between the formal and the real world in which a policy is formally introduced but not actually implemented and effective [21–23]. The adoption of concepts and policies followed by varying interpretations and symbolic implementation is one common reason for the occurrence of decoupling [24, 25]. Under such circumstances, a new policy seems to be implemented but may not have the intended consequences. The practices or constructs will be opaque, tenuous or even absent [24]. The issue of tokenism may also be related to the concept of decoupling [26].

Another related concept, namely recoupling, demonstrate how previously decoupled policies and practices become coupled, achieving substantive rather than symbolic compliance. Recoupling suggests that gaps between policy and practice may not be stable. Thus, policies and practices which were decoupled might become coupled and in some cases a brand new understanding emerges. Recoupling has been defined as the reversed process of decoupling. However, little is known about how recoupling can be promoted to diminish the gap between the formal and the actual [27, 28].

### Rationale and aim

Public health care has been associated with inertia when coming to change processes [29]. The implementation of soft care concepts and methods, such as PCC, has proven to be complex and difficult, which might lead to suboptimal impact [30, 31]. Some stakeholders may, according to Ocloo et al. [26], practice tokenism, i.e. making symbolic efforts to gain legitimacy when claiming the use of patient-involving methods. To enable the implementation of PCC, a prerequisite for reaching substantive rather than symbolic changes is to achieve congruence across organisational levels in the understanding of the concept of PCC. Consequently, to achieve increased involvement of patients and their families in the care process through PCC, the values and principles that underly the new practice, descriptions of essential functions as well as operational definitions need to be clear [32]. Thus, the aim of this study was to explore the congruence of managers’ perceptions and understanding of various aspects of PCC across three organisational levels in one health care region in Sweden in terms of coupling, decoupling and recoupling.

## Methods

This study is part of a larger project (*Implementing person-centred care: process evaluation of strategies, leadership and health economy*, IMPROVE), in which the implementation of PCC is studied in an observational case study with six embedded health care units aiming to increase the knowledge about implementation of PCC from different perspectives in a real-world context. The implementation of PCC was initiated and run by the studied health care organisation without any influence from the researchers. This qualitative study explored aspects of the process of spreading knowledge and understanding of PCC across a health care organisation, captured at the politician and management level.

### Setting

The IMPROVE project was conducted in a health care region in mid-Sweden with approximately 278,000 inhabitants in an area of 28,000 km^2^. All health care regions in Sweden (*n* = 21) have regional autonomy. Hence, the regional autonomy embraced six hospitals, of which two were emergency hospitals and approximately 30 primary health care units. As with health care services in general in Sweden, all hospitals and primary care units are publicly funded.

In 2015, a policy regarding “increased participation in the health care services for patients, relatives and patient and user organisations” was adopted in the studied health care region by the regional assembly. The policy was embodied in the form of PCC in the health care region’s politic strategic plan and steering document for the period 2016–2020.

The region chose to adopt an approach to PCC developed by the University of Gothenburg Centre for Person-centred Care (GPCC) [33]. According to this approach, PCC is considered an ethical standpoint that guides actions and involves three key concepts: initiating the partnership, working the partnership, and safeguarding the partnership. Initiating the partnership is done through listening carefully to patients or relatives’ narratives to understand resources, abilities and personal wishes and is a prerequisite for PCC. Working the partnership entails shared deliberation and agreements on, for example, medical investigations, treatments and responsibility for self-care that are documented in a health plan. Safeguarding the partnership entails documentation of the mutually agreed health plan to support continuity of care. The health plan should be updated when relevant.

Representatives from the health care region’s Department for Healthcare Development together with the CEO of the health care organisation developed the health care region’s support strategy for the implementation. The annual report from 2016 on the work for enhancing PCC practice described three core activities: gain legitimacy for implementing PCC, dissemination of knowledge and provision of support to middle managers, leaders and staff (Table 1). The strategy emphasised that the implementation efforts were aimed at making clinical practice *more* person-centred, as it was important to underline that much of the provision of care and treatment was already person-centred to a varying degree.

**Table 1:** Core activities in the health care region’s implementation strategy and description of activities

Core activity	Description of core activity
Legitimacy for the work process	Created through the adoption of the policy and the health care region’s political strategic plan and steering document. The CEO of the regional health care system and the politicians in the steering board supported the process.
Dissemination of knowledge	A series of three full-day learning seminars was arranged by the Department for Healthcare Development. Frontline and senior managers identified participants among their employees to the inter-professional teams that took part in the seminars. Sessions were led by representatives from the Department for Healthcare Development, stakeholders in the health care region, external researchers and patient representatives. The seminars included: • Lectures about PCC based on the model suggested by the University of Gothenburg Centre for Person-centred Care [34]: its philosophical and ethical underpinnings, the three core components and research findings from evaluations of PCC, e.g. shorter length of hospital, increased quality of life and relief of symptoms. • Presentations by representatives from a number of the health care units in the region and other stakeholders about how PCC could be practiced. • Lectures about methods for quality improvement, e.g. use of PDSA cycles. • Lectures about how E-health applications could support PCC. In addition, each seminar day included workshops with time to share reflections and experiences within the teams regarding their understanding of PCC, how it could be practices and implemented at the own unit.
Support to managers, leaders and health professionals	Senior managers and representatives from the health care staff unions discussed supportive efforts with managers and health professionals during the implementation of more PCC.

The principal activity directed towards the frontline and senior managers and staff was the dissemination of knowledge. There were no executive decisions; instead, soft management control to encourage rather than to push for the change was processed. Senior and frontline managers from each department and unit were invited to attend learning seminars together with a team of health professionals. Each health care unit then developed and ran its implementation strategies. No departments or units were explicitly forced to participate in learning seminars or to initiate the implementation of PCC even though all were expected to make such efforts. The unit received no extra funds and costs for the implementation were to be accommodated within the regular budget.

### Participants

The health care units (*n* = 11) participating in the first series of learning seminars conducted in 2016 were asked to participate in the IMPROVE study. We sought to recruit units that represented both in- and outpatient care at a secondary and primary care level and with a geographic spread over the region. This approach resulted in the inclusion of six health care units representing renal, primary, psychiatric and geriatric care.

For this study, data were vertically collected at three organisational levels in the health care system to detect coupling, decoupling and recoupling in descriptions of PCC. The organisational levels selected were based on the management roles of perceived senders, messengers and receivers [34, 35]. A sender was considered as one who initiates PCC (e.g., a stakeholder such as a chairperson in a steering board or a politician in the health care region). The messengers were recruited from senior managers regarded as mediators of decisions made by the health care region’s board. A typical receiver was a middle manager in the operating core, such as hospital clinics and wards [36].

### Senders – politicians and documents

Three politicians involved in health care issues were asked to participate and all accepted. They represented both men and women and their experience as politicians varied between 2 and 35 years. In addition, the document “Policy about increased participation in the health care services for patients, relatives and patient and user organisations” decided in 2015 and the region’s strategic plans decided by the regional political assembly for 2016–2019 and 2017–2020, also constituted part of the senders. Two of the senders participated a half-day each in the learning seminar.

### Messenger - senior management

Seven leading officials in the health care region’s administration or officials at central positions for the implementation of PCC were asked to participate. Two of these did not respond despite reminders sent by e-mail. The five officials that agreed to participate were of both sexes and their professional experience ranged from 20 to 43 years. Two representatives of the messengers managed the implementation at the regional level and arranged the learning seminar series. Two other messengers participated during limited parts of the series.

### Receivers – frontline managers and senior managers

All frontline and senior managers (*n* = 13) at the 6 units included in the IMPROVE study agreed to participate. They were both men and women and had from 9 to 45 years of work experience in the health care sector. Of the 13 receivers, 10 participated in the series of three full-day learning seminars, one in parts of the series and two not at all.

### Data collection

Semi-structured face-to-face interviews were conducted between March and December 2017 at the respondents’ workplace. An interview guide (see Additional file 1) was developed specifically for this study with questions about a) descriptions and characteristics of PCC, b) perceived external and internal sources of PCC and its spread, c) reason to implement PCC, d) expectations from implementing PCC and e) strategies used to implement PCC. The participants were asked to schedule 30 min for the interview and the interviews lasted between 21 and 46 min. The three first interviews were conducted by MT and EC while the remaining interviews by MT alone. All interviews were digitally recorded. Prior to the study, approval was granted by the regional ethical review board in Uppsala, Sweden. Informed consent was given individually by all participants.

### Analysis

The analysis was based on the empirical data collected in the interviews, i.e. we did not interpret reported perceptions and understanding in relation to any particular definition of PCC. The transcribed interviews were analysed in Microsoft Word using deductive qualitative content analysis [37] based on the study questions (a-e). Initially, a thorough reading of the transcripts was performed to acquire an overall picture. Next, an unconstrained matrix was developed. The interview data about the reasons for implementing PCC and expectations of the implementation could not be separated from one another and were thus merged into one category. Within the bounds of each category in the matrix, data were inductively coded and grouped into subcategories.

The codes were labelled by organisational levels (sender/messenger/receiver) to identify the levels represented in each subcategory. If two, or all three organisational levels were represented in a subcategory, this was considered as coupling regarding the topic for the subcategory whereas the lack of representation was considered de-coupling.

The analysis was led by MT and performed in collaboration with EC. Both MT and EC have experience of research within the field om implementation, EC in health care organisation, and from clinical work as a physiotherapist (MT) and nurse (EC). MT and EC individually and independent of each other coded and made a preliminary analysis of five interviews representing the three organisational levels. The analyses were compared and discussed to reach the most credible analysis and interpretation of the findings, and consensus was then reached about how to continue the process. MT continued the analysis which involved continuously going back and forth between the whole and the parts of the empirical data and discussion with EC. In addition to EC and MT, discussion about a preliminary analysis also involved LW who has experience of implementation research and clinical practice as a nurse. Preliminary findings were presented at one seminar for an extended research group including several researchers involved in the IMPROVE project and an open seminar at Dalarna university at which several of the study participants took part. During these seminars topics were discussed that contributed to further development of the analysis. Altogether, the authors’ discussion of the preliminary findings the comments from the seminars was helpful in our ambition to achieve the most credible analysis and interpretation of findings [37].

## Results

The deductive analysis, based on four predefined categories and inductively created subcategories, is presented in Table 2 and the text below. For each category, findings from the senders, messengers and receivers are shown separately.

**Table 2:** Categories, subcategories and their representation in the three organisational levels

	Sender	Messenger	Receiver
**Descriptions of PCC**			
Patients as persons with equal value	•	•	•
Involvement and co-creation	•	•	•
Organisation of work		•	•
**The sources of PCC**			
Dissemination from university, government agencies, society and other origins	•	•	•
Dissemination from internal units from above and the side	•	•	•
Ambiguity on the decision to adopt		•	•
Dissemination of a new label only		•	•
**Motives for implementing PCC and expected effects**			
Improved patient participation	•	•	•
Good care and satisfied patients	•	•	•
Reducing or redistributing health care costs	•	•	•
Improved work environment		•	•
Confirmation of current values			•
**Strategies to disseminate and implement PCC**			
Activities and actions to support change	•	•	•
Featuring existing routines and methods	•	•	•
Stimulate reflection and a more profound understanding		•	•
No action needed		•	•

### Descriptions of PCC

The categories’ descriptions of PCC capture the participants’ perceptions of the PPC concept and its distinctive features.

#### The senders

The senders described PCC as a model or approach that not only focused on the disease but the person as a whole and also meant a “small shift in power” in favour of the patients. Above all, the politicians focused on the patients’ experiences of the care provided by health care professionals. They presented a new role for patients, which entailed patients being involved in planning and evaluation, influencing their care and sometimes have a leading role in the care process.

PCC was further described as specific actions performed by the staff, such as listening to patients’ problems, co-creation of care and documenting agreements.

#### The messengers

Consistent with the senders, the messengers viewed PCC as an approach in which health professionals and patients are equal partners in a hierarchical system and where the partnership serves as a core component. Most prominent was the focus of the staff’s specific actions, which involved listening to the patients’ narrative, making agreements about the care and providing care that met the patients’ individual needs. Less attention was given to the patients’ experiences of the care provided.

In addition to the categories that concurred with those of the senders, the messengers’ description of PCC covered aspects of how work was organised. This description included how time and work tasks were allocated, suggesting that more of the staff’s time should be spent with patients and that the staff should perform work tasks that better engaged their level of competence.

#### The receivers

The receivers emphasised PCC as an approach and as a relationship between staff and patients entailing equal partnership with different, but equally valuable knowledge, and with shared responsibility. The health care professionals had to “let the patients in” and provide knowledge and support that enabled the patients to participate actively in care and treatment, e.g. handle their peritoneal dialysis or diabetes self-care. Patients should be seen as capable and resourceful, which would encourage the staff to have confidence in the patients’ ability. Metaphors from team sports were used to describe the role of the patients in PCC.

*“We are a team together with the patient and the patients are the key players in the team. Previously, we worked with the patient as a spectator.”*

It was stated that the care should be based on the patient’s needs, goals and life situation and this was illustrated by examples, such as adaptation of time points for meals and showering based on patients’ wishes at inpatient units and time intervals between check-ups for patients with diabetes in primary care.

In addition, similar to the view of the patient as a valuable source of knowledge, it was emphasised that the contribution of knowledge and experience of all the health professionals, regardless of coming from the physician or assistant nurse, were equally important.

Representatives of the receivers also proposed that PCC did not mean anything new and they, in line with the messengers, referred to issues linked to the allocation of work tasks and utilisation of competence, expressed as:

*… .that is that one should work with the patients, the relevant person for the relevant task. We should not have well-trained staff standing in the kitchen or cleaning beds; rather, they should only work with the patients’ care.*

### The sources of PCC

The category *sources of PCC* captures the participants’ view of what they perceived as internal and external sources from which the PCC concept was conveyed to their region and unit. The category further describes their knowledge and perception of decisions and official documents about the implementation of PCC.

#### The senders

The senders described rather vague, external sources to PCC, such as “movements that have existed in society for a long time” and an official national investigation of patients’ influence on their care but also ongoing research at a specific university as a specific source. The Health Care Services Office and the policy document for increased patient participation were described as internal sources. Other official documents mentioned were the region’s strategic plans decided by the regional political assembly and the health care region’s action plan. It was set forth that the four health care divisions in the region had started to enter PCC in their scorecards and action plans, implying that the concept was being integrated into several documents.

#### The messengers

Representatives of the messengers named a specific researcher at a specific university as the source but ‘speculations’ about the Swedish Association of Local Authorities and Regions and the National Board of Health and Welfare as a possible source for the concept were also expressed. The internal sources were “from above” or from the regional health care system’s CEO. The messengers stated the policy for patient participation as an official document but also referred to legislation and national policy. Other messengers did not know of any official document. They mentioned the lack of management control of the implementation, describing it as an “affirmation”from stakeholders and not as a clear decision to implement PCC. A few messengers emphasised that it was only the ‘label’ PCC that was new, and this label was being applied to already established ways of working.

#### The receivers

In line with the other participants, several receivers suggested a specific university as the external source but also mentioned the Swedish Association of Health Professionals (the trade union for nurses, midwives, biomedical analysts and radiographers), and further as a “fashion in Sweden’s health care industry”. Internal sources the receivers mentioned were similar to those of the senders and messengers, and additionally their own unit’s practice development staff or the frontline manager at an adjacent ward.

The receivers described the information and documents from the learning seminars as the official documents for the implementation of PCC. Other receivers referred to the health care region’s policy in general and legislation about patients’ participation in their health care. In addition, several receivers mentioned a lack of clarity about whether any decision regarding implementation was taken or whether this was more of an ‘orientation’. Some receivers were also concerned that the lack of an overarching plan could lead to significant differences between departments.

In agreement with the messengers, a few representatives suggested that only the label itself (i.e. PCC) was a novelty.

*… PCC is the formal name, but everyone knows that here in Sweden we have worked in this way for several years. But now, there is a desire to formulate it in writing and give it a name. But it is not new, so it is not that we start a whole new way of working.*

### Motives for implementing PCC and expected effects

The category *motives for implementing PCC and expected effects* includes five subcategories that focus on the motives for enhanced person-centredness interwoven with the expected effects of the implementation of a PCC approach.

#### The senders

The senders took the patients’ perspective, advocating the demands from patients and their organisations for more power and greater participation in care as motives for the implementation. They also suggested that the health care region, as well as the whole country, “is perhaps a bit behind when it comes to this approach”. The senders expected health professionals to gain improved ability to share knowledge, to include the perspective of patients and feel comfortable when involving patients and not afraid to transfer power to them.

*So we expected that our employees would feel more secure in their approach towards patients and may not be afraid to leave some of ... yes ... maybe power to the patient.*

Generally, equal and more efficient care of good quality was expected. More specifically, a few senders anticipated positive economic effects and shorter length of hospital stay as motives for the implementation.

#### The messengers

The messengers expected that patients would be more involved in their own care and that the healthcare system did not ‘take over’ tasks that the patients could do themselves.

Like the senders, the messengers described expectations related to the quality of care, improved patient safety, healthier and more satisfied patients and relatives. Expected effects were also recounted in terms of reducing or redistributing health care resources, which involved more time spent with patients instead of on administrative tasks and “as a factor when dealing with financial conditions”.

The messengers also expressed problems with the work environment, such as nursing shortage and the need for temporarily employed physicians, as motives to implement PCC. PCC was expected to improve the working conditions of health professionals.

#### The receivers

The receivers anticipated PCC to improve the patients’ ability to increase their care involvement and manage their treatment better. Like the views of the senders and messengers, the receivers pointed out motives related to improved patient safety and quality of care.

Several of the receivers put forward issues related to the allocation of resources and had strong expectations for shorter length of stay at hospital. In addition, most of the receivers discussed the working environment as reasons for the change and expected effects. PCC was anticipated to influence staff such that they would enjoy their work time more, improve satisfaction with work, cope better with their workload and not resign.

In addition to the motives described by the senders and messengers, the receivers expressed that the consistency with current values was a reason for the implementation. The implementation of PCC maintained and enforced already ongoing initiatives that were person-centred. PCC was described as easy to implement because it was consistent with, or close to, the present clinical practice.

### Strategies to implement and disseminate PCC

The category *strategies to disseminate and implement PCC* describes how PCC was realised at both the health care region and health unit level. The category also describes the various strategies used to support the change towards more PCC.

#### The senders

The three-day learning seminar series was seen as crucial for dissemination of knowledge about PCC to support the implementation at the clinical departments. Moreover, the senders described actions to support the change towards a more PCC involving the adoption of the policy on patients’ participation in care, which was considered conceptually close to PCC. PCC was also used in operational plans and balanced scorecards. In addition, establishing a dialogue between senior management and patient and user organisations was considered to provide a “systematic contribution” in this direction.

The senders also suggested the use of the already existing structures and routines of the health care region that were considered person-centred as components of the implementation strategy. These structures and routines included the patients’ online access to their health records, the opportunity to write self-referrals and numerous eHealth initiatives.

#### The messengers

Representatives of the messengers emphasised the strategic decision not to formulate an executive decision on the implementation of PCC and pushing for change, but rather focusing more on supporting clinically based initiatives with a PCC approach and existing change agents and opinion leaders. The learning seminars were therefore offered to teams from each unit to help initiate the process, increase knowledge, exchange experiences and for reflection. Participation was voluntary and each unit was thereafter expected to run its own change process.

In line with the senders, the messengers proposed region-wide structures, such as a list of patients who were willing to share their experience-based knowledge. Moreover, clinical working models that were considered person-centred were described. Further strategies involved dialogues between managers at various levels to show commitment to the change process.

A change towards PCC was perceived to imply individual and collective development based on a more profound understanding that required intensive reflection and careful consideration. The difficulty of ordering such a change was expressed by one messenger in the following terms:

*This is no directive that can come from the top management and be imposed on our departments without the staff having reflected on what PCC means. This is because we do not believe that a document can describe what PCC is. We believe that one must feel what it can mean in one's clinical context.*

Occasionally, messengers described that only a few, or even no, measures were needed as the care was already person-centered.

#### The receivers

Several of the receivers’ representatives referred to their participation in the three-day learning seminar series as crucial for their continued efforts. Health professionals participating in the seminars were assigned responsibility for running the process together with the frontline managers. Strategies for supporting the change involved disseminating information and knowledge during different meetings, providing peer supervision and coaching, emphasising the importance of the change by asking for a PCC approach in the staffs’ clinical practice, jointly reflecting on good and bad clinical examples, and talking about PCC in employment interviews. Further strategies involved to define activities related to the implementation of PCC in the department’s action plans and organize work to enable the staff to work with the implementation. In addition, to make PCC – by many perceived as ‘fuzzy’ and ‘diffuse’ – to something more tangible was described as important.

Several existing components of the care such as patient education, self-management programmes and care coordinators were described as person-centred, and the implementation strategies included the intention to continue to support these activities and sometimes develop them further. New components or routines that were planned or already launched to promote more PCC were put forward. These initiatives included teamwork and better routines for admitting patients. Moreover, to educate staff in, and start to use, motivational interviewing during clinical encounters was seen by several of the receivers as an important measure in implementing PCC.

In line with the messengers, the change towards PCC was described to involve individual development and change, expressed as ‘to find it in everyone’. In addition, some representatives occasionally reported that they already provided PCC while others equated PCC with different care concepts currently in use. Below, the perceived fuzziness and diffuseness of the PCC concept along with the ambition to continue to improve the care process is illustrated.

*It’s called PCC now. Yes, we have had the patient in the centre... now PCC is the trend. But it's about finding the best path for the patient no matter what you wish to call it. And we work with that in many different ways. Other departments have contact nurses and various kinds of care coordinators. That is also PCC because you facilitate the patient’s path. So, I think it's unfortunate to call it different things because this makes people confused.*

## Discussion

**Fig. 1:**
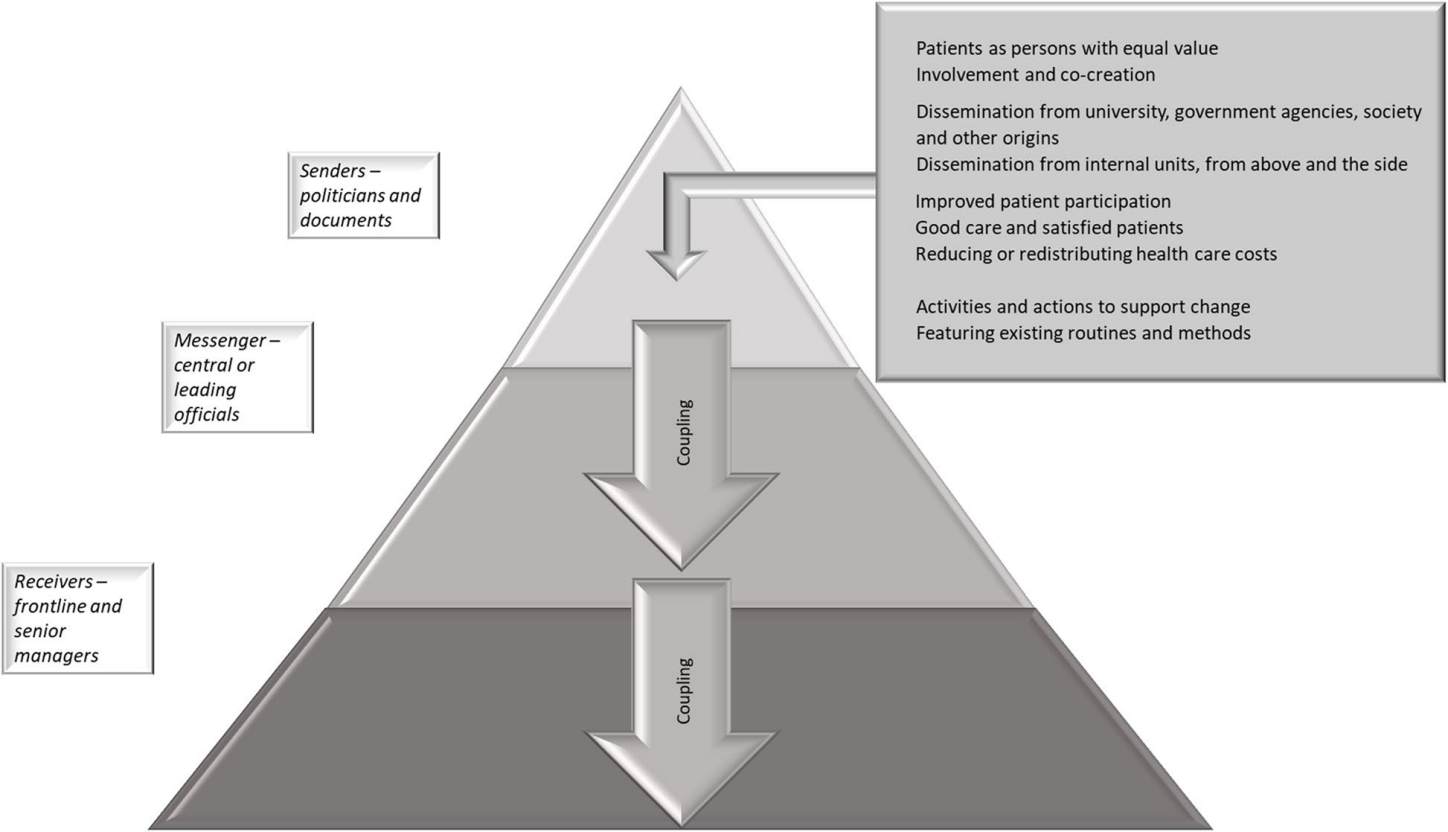
The findings reflect coupling across the three organisational levels, (i.e. the senders, messengers and receivers) concerning most aspects of descriptions of PCC, the sources of PCC, motives for implementing PCC and expected effects, and strategies to disseminate and implement PCC

**Fig. 2:**
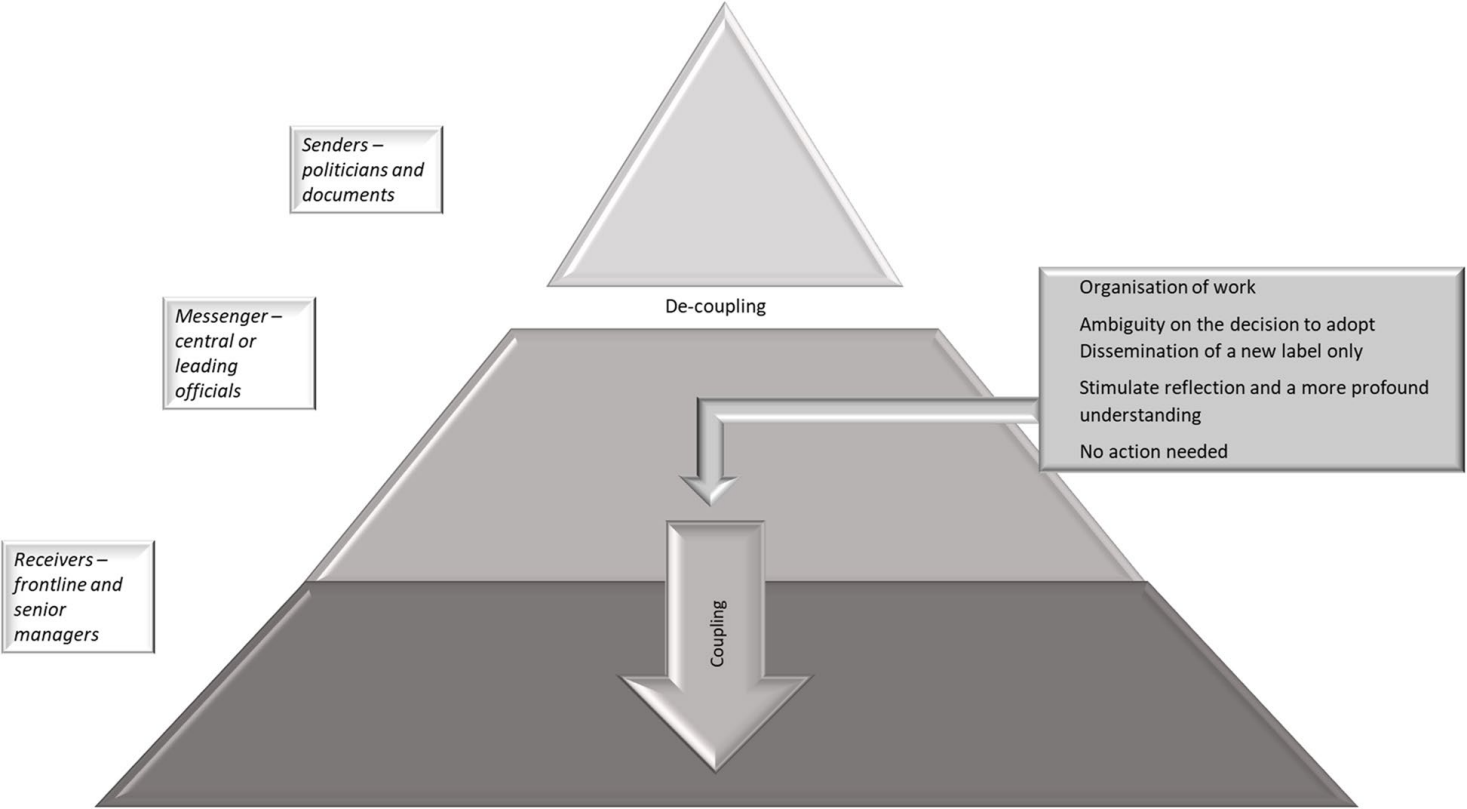
The findings reflect some de-coupling between the senders and the lower organisational levels (i.e. messengers/receivers) concerning a few aspects of descriptions of PCC, the perceived sources of PCC, motives of the implementation and expectations of PCC effects and strategies for supporting the implementation and an expanded perception of these aspects of PCC

**Fig. 3:**
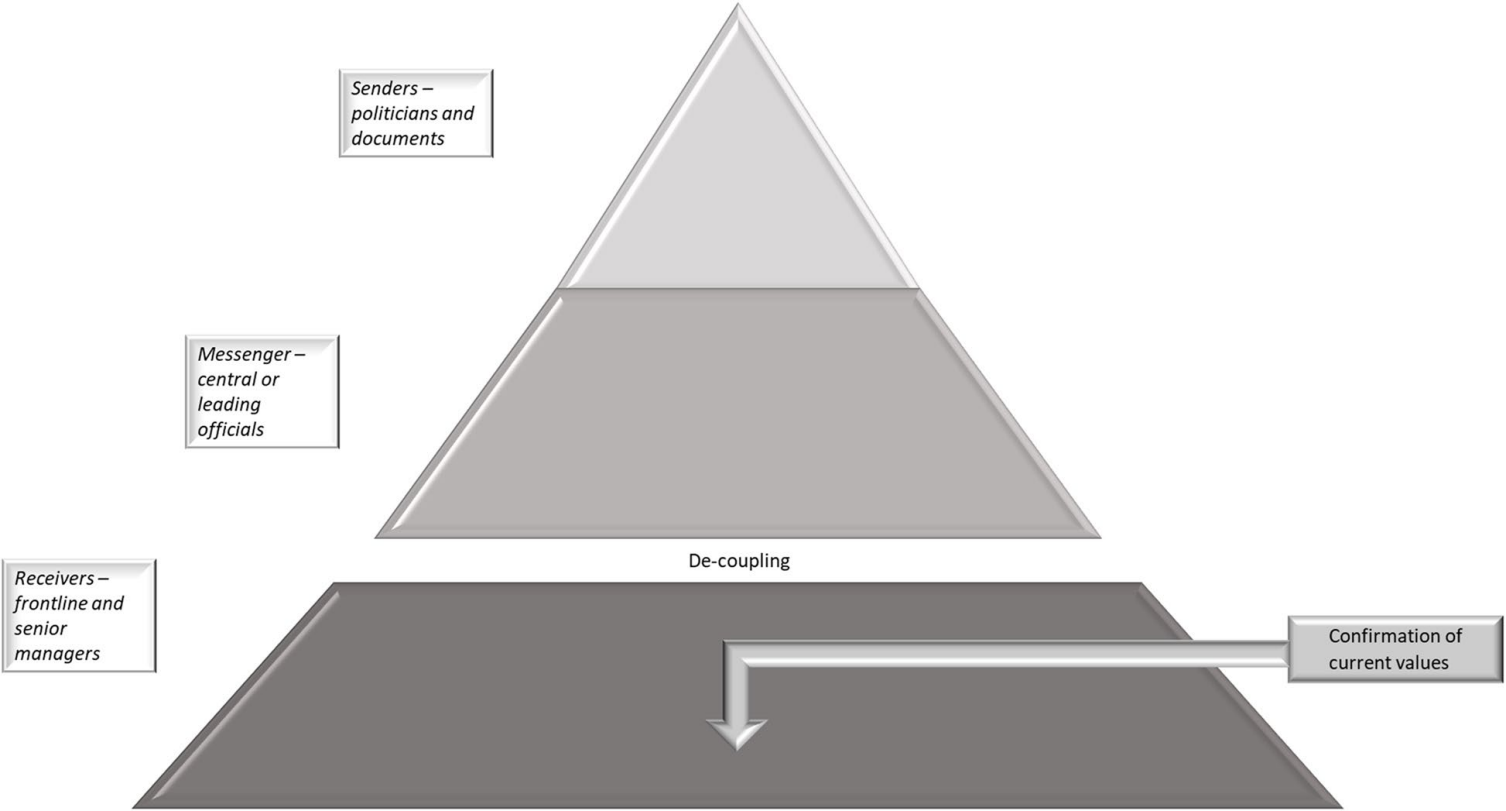
The findings reflect de-coupling between the senders/messengers and receivers concerning one aspect of expectations of PCC effects and strategies for supporting the implementation and an expanded perception of this aspect of PCC

We analysed congruence across three organisational levels in a regional health system concerning the descriptions of PCC, the sources of PCC, motives for implementation, and strategies used to implement PCC and expectations of PCC effects. By exploring these aspects at three organisational levels, we wanted to increase the knowledge on how the perceptions and understanding of a policy regarding the participation of patients and relatives in health care services in the form of PCC, shaped the conditions for PCC in clinical practice. As illustrated in Fig. 1, our findings indicate that the three organisational levels were congruent concerning some of the aspects explored in the study, suggesting that coupling between policy and practice was evident. However, as illustrated in Figs. 2 and 3, also incongruences were identified, indicating de-coupling. This de-coupling represented a gap between the restrictive perceptions of the explored aspects regarding PCC among politicians and in governing documents, compared to the broader view among the senior management and middle and frontline managers. Re-coupling was not identified at this stage of PCC implementation.

Congruence across the three organisational levels regarding several aspects of the descriptions and perceptions of PCC was identified. Representatives of the senior management (i.e. the messengers) had originally suggested implementing PCC in the health care region and prepared the policy adopted by politicians (the senders). Representatives of the messengers were in charge of the region’s implementation strategy and thus good concurrence in fundamental aspects between the two highest organisational levels (i.e. senders and messengers) could be expected. Concerning the congruence with the frontline and middle managers, the findings indicate that the learning seminars, which were a core component of the region’s implementation strategy, played an important role. During these seminars, GPCC’s conceptualisation of PCC (patients’ narrative, partnership, documentation) was introduced and this conceptualisation permeated the managers’ descriptions of PCC. Research findings, including better relief of symptoms and shorter hospital stays, reported in studies where this conceptualisation of PCC had been applied were presented at the seminars, consistent with motives and expectations found at all management levels. Moreover, the learning seminars were considered the most prominent support for implementation and documentation from these seminars was referred to as the official description of the new practice. Research about the impact of such seminars on managers’ beliefs, perceptions and knowledge is scarce, but educational meetings have been reported to influence nurses’ knowledge and attitudes [38]. In addition, a systematic review of implementation strategies that promote management decisions in health care indicates that workshops along with other strategies may improve managers’ knowledge, skills and attitudes [39]. However, both the quality and quantity of the studies included are limited. Nevertheless, in the present study the learning seminars seem to have contributed to a way of describing and perceiving aspects of PCC that created coupling across the organisational levels coherent with the political ambition.

A number of subcategories were not represented at all three organisational levels, suggesting some decoupling across the levels. One specific manifestation of the lack of decoupling across the organisational levels was the perception of the level of person-centredness in the existing practice, and subsequently, the relevance of further implementation efforts as shown in Fig. 2. Whereas the politicians and governing document called for change, representatives of the lower organisational levels considered PCC to be an already established practice and accordingly, no actions for change required. The varying views on the need for change may partly be attributed to the perceived fuzziness of the concept and the difficulty to define the content of PCC and what it entails at the practical level. Knowing whether a practice is person-centred might be challenging. Indeed, a recent study on the implementation of PCC reported that health professionals claimed to be practising PCC, although experts in the field disagreed [40]. Another study points out the challenge and importance of health professionals adopting both the theoretical assumptions and procedures of a PCC intervention [41]. Furthermore, because no generic measure exists to assess the level of person-centredness [42], moving towards PCC without tools that assist in creating a shared view of current practice and monitor potential progress is challenging.

Another manifestation of the lack of congruence was that the descriptions of PCC, as reported by senior management and middle and frontline managers, expanded beyond the conceptualization adopted by politicians. Unlike the politicians and the regional documents, PCC was seen as a concept that included issues related to the aspect of how time and work tasks were allocated and the use of the health professionals’ competence (Fig. 2). In addition, motives and expectations for implementing PCC involved improvement of the present staff situation, which was often overworked and undermanned and with a high turnover of nurses at some departments. These incongruences across the organisational levels can also be interpreted as a consequence of the lack of clarity about the PCC concept and its application in clinical practice. Previous research suggests that managers are motivated to implement change if they perceive that the implementation is important and of organisational relevance [39]. Thus, the perceived vagueness of how PCC should be applied might have led some managers to broaden the concept to include aspects that were important to them in their day-to-day activities, such as issues related to staff shortage.

Another reason for the different views of whether PCC was being practised could be that the units included in this study might not have adequately represented the region’s actual standard concerning the level of person-centredness. The studied units chose to, as illustrated in Fig. 3, invest in implementation and the respondents highlighted the similarity between the present practice and PCC as a reason for initiating the implementation. It is therefore reasonable to assume that some of these units had already, at least partly, integrated PCC in practice and could be considered early adopters [43] of the PCC approach.

### Strengths and limitations

To our knowledge, this is the first study on the spreading of a general conceptualisation of PCC in real-world settings within a health care region. Previous studies have described the implementation of specific person-centred interventions (e.g., a person-centred process of carer assessment and support) [44] or PCC within a particular context (e.g., dementia care) [45]. When interpreting the current findings, some limitations must be taken into consideration. Using a case study design with six embedded units, we have investigated the implementation of PCC in one health organization. This design is well suited to explore contemporary phenomenon in real-world contexts [46], even though the single case approach does not allow for contrasting of findings from other cases. We included six health care units in the IMPROVE-project as these units represented both primary and secondary care, inpatient and outpatient care, had geographical spread and were considered to represent sufficient variation to provide rich data to reach the project’s overall purpose. In terms of saturation of data in this specific study, all units included in the IMPROVE project were represented on messenger and frontline level, therefore no additional persons were relevant to ask for participation. When it comes to inclusion of politicians, those included were the persons most involved and informed about the PCC implementation, although inclusion of additional politicians might have contributed with further information. With regard to saturation in individual interviews, the participants were asked to book 30 min for the interviews as this was considered reasonable based on the research question and the fact that many participants had very busy schedules. The length of the interviews varied as some were not very involved and did not have much information to provide while other were more engaged and contributed with data at such level of detail that it was beyond the purpose of this study. Overall, considering saturation of data with regard to both number of participants and individual interviews, we judge that the dominant and most relevant perceptions and understanding of PCC across the three levels were captured.

The cross-sectional design does not acknowledge implementation as a dynamic process and presents difficulties in capturing recoupling. A longitudinal design had increased the opportunity to explore such aspects but was beyond the scope of our study. Finally, this study presents the perspective of the managers across the health care region whereas the perspectives of the health professionals and patients are lacking. However, the IMPROVE projects seeks to increase the understanding of PCC and its implementation in depth from several perspectives and the perspectives of the health professionals and patients have so far been covered in two publications [47, 48] and future publications will present findings on e.g. health economy and implementation strategies. To sum up, we suggest that the findings from this study could be relevant in the spread and implementation of PCC also in other health care contexts.

## Conclusions

In conclusion, using the concepts of coupling, decoupling and recoupling, an outcome of full coupling appeared to be impossible to achieve, some decoupling was inevitable and recoupling might be a prolonged effect not yet seen. The learning seminars, which were the core component in the region’s support strategy, seemed to have promoted common understanding and coupling to some extent. Full coupling, i.e. the idealistic outcome of management control, was difficult to achieve because of the fuzziness of definitions, the challenge to achieve a common view of the actual level of person-centredness and consequently the need for further implementation efforts.

## Supplementary Information

**Additional file 1.** Interview guide.

## Data Availability

The dataset generated and analysed during the current study is not publicly available as that would compromise participants’ consent but are available from the corresponding author on reasonable request.

## Abbreviations

PCC: Person-centred care (PCC)
IMPROVE project: Implementing person-centred care: process evaluation of strategies, leadership and health economy
GPCC: Centre for Person-Centred Care, University of Gothenburg, Gothenburg, Sweden
