# Supplementary material for: A comparison of three organisational levels in one health care region in Sweden implementing person-centred care: coupled, decoupled or recoupled in a complex organisation
Source: BMC Health Serv Res. 2022 Feb 14;22:196. doi: 10.1186/s12913-022-07548-8 (PMC8842547; doi:10.1186/s12913-022-07548-8)
Supplement: Supplementary file 1 — Additional file 1. Interview guide. [file 12913_2022_7548_MOESM1_ESM.pdf]

Interview guide

How would you describe the person-centered care “concept” that you implement? What are the main characteristics of the concept?

Where did the concept of PCC and it’s spread in the region come from?

Why do you want to introduce the model?

How have you decided to implement PCC?

What expectations on the implementation of PCC do you have?
